# Nasogastric tube after oesophagectomy and risk of anastomotic leak: a Nordic, multicentre, open-label, randomised, controlled, non-inferiority trial

**DOI:** 10.1016/j.lanepe.2025.101411

**Published:** 2025-07-31

**Authors:** Jakob Hedberg, Joonas Kauppila, Eirik Kjus Aahlin, David Edholm, Gjermund Johnsen, Jan Johansson, Pernilla Lagergren, Mats Lindblad, Fredrik Lindberg, Olli Helminen, Per Löfdahl, Dag Tidemann Førland, Mads Vikhammer, Pieter de Heer, Magnus Sundbom, Eva Szabo, Oscar Åkesson, Magnus Nilsson, Albert Nilsson, Michael Achiam, Tom Mala

**Affiliations:** a Dept of Surgical Siences, Uppsala University, Uppsala, Sweden; b University of Oulu and Oulu University Hospital, Oulu, Finland; c Faculty of Health, Institute of Clinical Medicine, University of Tromsø, Norway; d Department of Surgery in Linköping and Department of Biomedical and Clinical Sciences, Linköping University, Linköping, Sweden; e Department of Gastrointestinal Surgery, Norwegian University of Science and Technology, Trondheim, Norway; f Department of Surgery, Skane University Hospital, Lund, Sweden; g Department of Clinical Science, Intervention and Technology (CLINTEC), Karolinska Institutet and Department of Upper Abdominal Diseases, Karolinska University Hospital, Stockholm, Sweden; h Department of Molecular Medicine and Surgery, Karolinska Institutet, Stockholn, Sweden; i Department of Surgery and Cancer, Imperial College London, London, UK; j Department of Diagnostics and Intervention, Surgery, Umeå University, Sweden; k Department of Surgery, Sahlgrenska University Hospital, Gothenburg, Sweden; l Department of Pediatric and Gastrointestinal Surgery, Oslo University Hospital, Institute of Clinical Medicine, University of Oslo, Norway; m Department of Surgery, Örebro University, Örebro, Sweden; n Department of Biostatistics, Uppsala Clinical Research Center, Uppsala University, Sweden; o Department of Surgery and Transplantation, Copenhagen University Hospital, Copenhagen, Denmark; p Department of Clinical Sciences, Lund University, Lund, Sweden

**Keywords:** Oesophageal cancer, Oesophagectomy, Nasogastric tube, Anastomotic leak, Complications, Postoperative care

## Abstract

**Background:**

Oesophagectomy, a corner stone in curative treatment of oesophageal cancer, is a complex procedure with high complication rates. Postoperative gastric tube decompression is debated and some centres are abandoning routine nasogastric (NG) tube use. We hypothesised that postoperative NG tube removal is non-inferior to five days of NG tube decompression, with regard to the risk of anastomotic leak.

**Methods:**

In this open-label, non-inferiority randomised controlled trial across 12 hospitals in Sweden, Norway, Denmark and Finland, participants treated for oesophageal or gastroesophageal junctional cancer with oesophagectomy were randomly assigned (1:1) to no postoperative NG tube or five days of NG tube decompression. Anastomotic leak was the primary outcome and secondary outcomes included pneumonia and length of hospital stay. Analyses were performed on the intention to treat and per protocol populations and non-inferiority for anastomotic leak was defined as a risk difference below 9%. ISRCTN.com registration ISRCTN39935085.

**Findings:**

Between January 1st 2022 and March 27th 2024, 448 patients were randomly assigned, 217 to no postoperative NG tube and 231 to five days NG tube treatment. The mean age was 67.5 (standard deviation (SD) 9.8) years and 367 (81.9%) were males. Non-inferiority with regard to anastomotic leak for no NG tube decompression could not be shown with 48 patients (22.1% (95% confidence interval (CI) 16.8%, 28.2%)) having anastomotic leak compared to 35 (15.2% (95% CI 10.8%, 20.4%)) with five days of NG tube decompression, a risk difference of −7.0% (95% CI −14.4%, 0.00%), p_non-inferiority_ 0.30. In a Supplementary analysis, patients had a lower risk of anastomotic leak if postoperative NG decompression was used. Rate of other complications, e.g., pneumonia, were similar between groups. In a per-protocol analysis, the risk difference was −11.3% to the advantage of NG tube (95% CI, −19.1, −0.3%).

**Interpretation:**

We could not establish safety (increased risk of anastomotic leak) and therefore do not support omission of NG tube after oesophagectomy.

**Funding:**

This trial was funded by the 10.13039/501100002794Swedish Cancer Society and the Nordic Cancer Union.

**Research in context**

**Evidence before this study**

We searched PubMed from database inception to October 24th 2024 using the terms nasogastric tube and oesophagostomy. We found five retrospective trials of nasogastric (NG) tube use after oesophagostomy, two of which included a propensity score matched analysis. Four randomised trials investigating omission of NG tube use have been published, but the largest of these contain 150 patients in total. One of these trials, including 40 patients in total, showed increased leak-rates in the NG tube group and none of the trials have shown risks associated with abstaining from NG tube use. Therefore, many authors advocate moving away from using postoperative NG tubes, and this approach has been implemented in clinical practice.

**Added value of this study**

When moving away from a generally accepted clinical practice on the assumption of clinical equipoise, it is important to establish non-inferiority regarding risks such as anastomotic leak. Ours is the first adequately powered trial to this end and despite a non-inferiority level of −9% in risk difference we could not establish non-inferiority with a risk difference of 7.0% to the benefit on NG tube use.

**Implications of all the available evidence**

Since 2006, four randomised controlled trials have been published, and several reviews conclude that omission of NG tube is safe and beneficial for patients. We challenge this conclusion. Our trial could not show non-inferiority for omission of NG tube and more of the devastating complication of anastomotic leak was seen if NG tube was not used. This suggests that the discomfort of NG tube in the immediate postoperative period is a price worth paying. We therefore advocate the use of postoperative NG tube after oesophagectomy with gastric conduit reconstruction.

## Introduction

Oesophageal and gastroesophageal junctional cancers cause more than 500,000 deaths annually and are estimated to afflict almost one million individuals per year by 2040.^1^ Oesophagectomy is the mainstay of curative treatment, but associated with high complication rates, whereof pneumonia is the most common and anastomotic leak the most dreaded.^1^^,^^2^ Postoperative use of nasogastric (NG) tube for drainage and decompression of the gastric conduit is routine in most centers.^3^, ^4^, ^5^, ^6^ This is the last area of gastrointestinal surgery where routine NG tubes are still used postoperatively.^7^ The main rationale for the use of NG tube is to reduce risk of anastomotic leak and its consequences, and to reduce the risk of pulmonary complications from aspiration. Unfortunately, the NG tube is associated with significant discomfort for the patients^8^ and have risks of its own including aspiration. Some authors have therefore discouraged its use.^9^, ^10^, ^11^

Abstaining from NG tube use after oesophagectomy has been deemed safe in several smaller sample sized studies and trials and is, at least in the short term, a more comfortable routine for the patient. This practice has therefore been incorporated in post-oesophagectomy enhanced recovery programmes.^12^^,^^13^ However, modifying and changing a deeply rooted clinical practice should be based on adequate evidence of safety and data from adequately powered randomised controlled trials. We hypothesized that refraining from postoperative NG tube use is non-inferior to five days of NG-tube postoperatively, with regard to anastomotic leak and other early outcomes and we therefore launched the RCT kiNETiC (Randomized Controlled Trial- Ng-tube post-EsophagecTomy Complications)-trial. We chose to compare zero versus five days of NG tube decompression in order to give easily interpretable and clinically relevant data for practising surgeons. The aim of this trial was to provide the first high level evidence regarding the safety of abstaining from NG tube use after oesophagectomy.

## Methods

### Study design

A multicentre, randomised controlled trial with non-inferiority design was performed with anastomotic leak as the primary endpoint. Patients were included in 12 university hospitals in Sweden, Norway, Denmark and Finland, with ethical approval for each country. Trial registration and protocol can be accessed at https://doi.org/10.1186/ISRCTN39935085. A pretrial survey across the Nordic Upper Gastroinestinal Cancer Centres demonstrated that all study centres routinely used NG tube after oesphagectomy.^3^

### Patients

**Fig. 1:**
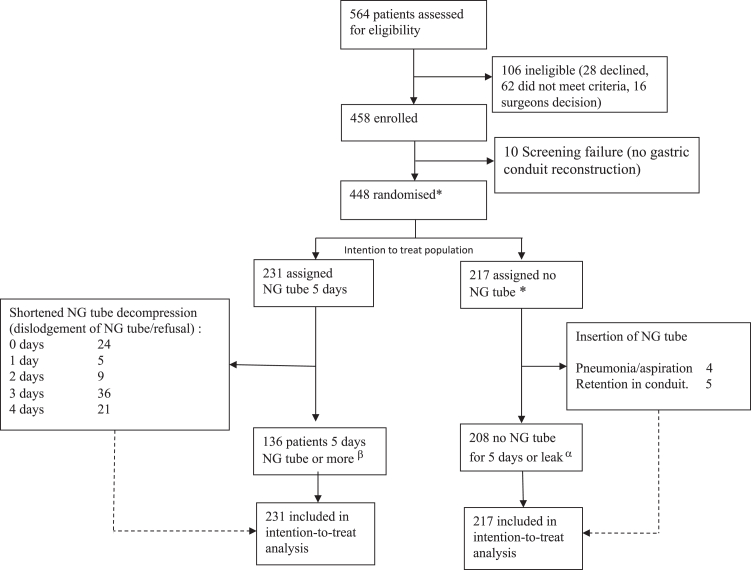
Trial profile. NG tube: Nasogastric tube decompression of the gastric conduit after oesophagectomy for cancer. ∗ Safety population was defined as the whole population which received the experimental intervention. α The per protocol intervention group was defined as all patients who had no NG tube. β The per protocol control group was defined as patients who were treated with nasogastric drainage for five days or more.

Patients above the age of 18 with histopathologically confirmed oesophageal or oesophago-gastric junction cancer (cT1a N+ or cT1b-4a any N; M0) that were considered technically resectable by the local tumour board were screened for inclusion and included after oral and written consent at the participating university hospitals. Patients were excluded if no resection was performed, other conduit types than gastric conduit were used (jejunal/colonic interposition), or other specified surgical technical reasons (e.g., non-routine anastomotic technique), Fig. 1. All randomised patients were defined as intention to treat (ITT) population. All patients randomised to the intervention (i.e., no postoperative NG tube) were considered the safety population and patients in the control group in whom the NG tube was extracted before day five were analysed separately for safety.

### Randomisation

Randomisation was performed by the surgeons involved accessing the web-based randomisation module. Patients were randomised 1:1 to i) no postoperative NG tube (intervention) or NG tube use five days postoperatively (control). Patients randomised to the intervention had their NG tube removed before leaving the operating theatre. An on-line baseline registration (DynaReg®, Stockhom, Sweden) was performed followed by permuted block randomisation stratified for sex, neoadjuvant treatment (yes/no), anastomotic site (thorax/neck) and centre. The surgeon or research nurse who accessed the randomisation module then informed the operating team of treatment allocation. No concealment of patients’ allocation was used.

### Procedures

All patients underwent oesophagectomy for cancer with gastric conduit reconstruction according to local routines as previously described.^3^ A senior oncologic surgeon was responsible for all procedures at all sites. Briefly, a gastric conduit was used for oesophageal reconstruction and the majority of patients underwent minimally invasive procedures after oncological neoadjuvant treatment, Table 1. For patients randomised to the experimental arm, no postoperative NG tube was used. In the control group, an NG tube was left in place for five days and handled according to local routines but was no less than 16 Charriere in size and with the drainage bag typically placed on the floor for siphoning suction. Postoperative regimes included early ambulation, with start of jejunostomy-based enteral nutrition or peroral feeding and chest x-rays according to local routines.^3^ At postoperative day seven, all patients underwent a computed tomography (CT) scan of the thorax and abdomen with peroral contrast. The radiologist assessing the CT scan was blinded to the study arm allocation.

**Table 1:** Baseline characteristics of 448 patients undergoing oesophagectomy for cancer.

	No NG tube (n = 217)	NG tube five days (n = 231)
Sex		
Male	173 (79.7%)	194 (84.0%)
Female	44 (20.3%)	37 (16.0%)
Age (SD, range)	68.0 (10.0, 24–84)	67.1 (9.7, 31–84)
ASA score		
1	13 (6.0%)	10 (4.3%)
2	113 (52.1%)	113 (48.9%)
3	87 (40.1%)	108 (46.8%)
4	4 (1.8%)	0 (0.0%)
Smoking		
Yes	21 (9.7%)	24 (10.4%)
No	106 (48.8%)	133 (57.6%)
Former	86 (39.6%)	74 (32.0%)
Unknown	4 (1.8%)	0 (0.0%)
Operation		
Ivor lewis	179 (82.5%)	194 (84.0%)
McKeown	38 (17.5%)	37 (16.0%)
Surgical access		
Open surgery	14 (6.5%)	15 (6.5%)
Hybrid MIO	81 (37.3%)	76 (32.9%)
Total MIO	122 (56.2%)	140 (60.6%)
Peroperative pyloroplasty		
Yes	34 (15.7%)	42 (18.2%)
Clinical T stage		
T1	7 (3.2%)	9 (3.9%)
T2	36 (16.6%)	42 (18.2%)
T3	152 (70.0%)	161 (69.7%)
T4a	22 (10.1%)	19 (8.2%)
Clinical N stage		
N0	108 (49.8%)	118 (51.1%)
N1	60 (27.6%)	59 (25.5%)
N2	41 (18.9%)	42 (18.2%)
N3	8 (3.7%)	12 (5.2%)
Clinical M stage		
M0	211 (97.2%)	217 (93.9%)
M1	6 (2.8%)	14 (6.1%)
Neoadjuvant treatment		
None	35 (16.1%)	42 (18.2%)
Radiochemotherapy	57 (26.3%)	69 (29.9%)
Chemotherapy	125 (57.6%)	120 (51.9%)
Center		
1	25 (9.2%)	29 (9.5%)
2	48 (22.1%)	50 (21.6%)
3	24 (11.1%)	28 (12.1%)
4	20 (9.2%)	22 (9.5%)
5	29 (13.4%)	30 (13.0%)
6	5 (2.3%)	5 (2.2%)
7	3 (1.4%)	5 (2.2%)
8	17 (7.8%)	12 (5.2%)
9	21 (9.7%)	21 (9.1%)
10	3 (1.4%)	6 (2.6%)
11	12 (5.5%)	9 (3.9%)
12	10 (4.6%)	14 (6.1%)
Country		
Sweden	118 (54.4%)	125 (54.1%)
Norway	65 (30.0%)	64 (27.7%)
Denmark	24 (11.1%)	28 (12.1%)
Finland	10 (4.6%)	14 (6.1%)

Ivor Lewis: two field thoracoabdominal oesophagectomy.

McKeown: three field thoracoabdominal oesophagectomy with cervical anastomosis.

Hybrid MIO: minimally invasive oesophagectomy (laparoscopy/thoracoscopy) in one operative field (abdomen or thorax).

Total MIO: minimally invasive oesophagectomy in both fields.

Baseline demographics, surgical procedures and neoadjuvant treatment.

### Outcomes

The primary outcome was proportion of anastomotic leak (yes/no). Secondary outcomes were leak graded on a three-level scale according to Low et al.,^14^ overall complications according to Clavien-Dindo,^15^ pneumonia according to Seesing et al.,^16^ length of hospital stay, length of stay in high-dependency unit as well as 30- and 90-day mortality. A clinical consultation with completion of an electronic case report form was performed six weeks postoperatively at which all outcomes were registered. Data on 90-day survival was retrieved from national death registries with 100% completeness.

Serious unexpected adverse events were to be reported to the sponsor within 24 h. An independent data safety and monitoring committee performed an assessment of the trial February 20th 2023, after inclusion of 207 patients, and deemed it safe and recommended continued inclusion.

### Statistical analysis

#### Sample size

A non-inferiority design was chosen with a non-inferiority threshold of 9% difference for the occurrence of anastomotic leaks. The non-inferiority threshold was selected based on the variability in leak rates in the Swedish quality registry for oesophageal cancer over the recent decade.^17^ Based on a leak rate of 17% in the control group, a power (beta) of 80% and a significance (alfa) of 5%, this would require 216 patients in each group or 432 patients in total. To account for unexpected withdrawals and loss of follow-up, we aimed at randomising 450 patients.

#### Analyses

First, all analyses were made on the intention to treat study population. Non-inferiority regarding anastomotic leak was assessed with a difference in proportions using a stratified Miettinen-Nurminen one-sided test with Mantel-Haenszel weights. The following four strata were used: women aged <70 years, women aged ≥ 70 years, men aged < 70 years, and men aged ≥ 70 years and was chosen to increase power since the frequency of the outcome was expected to depend on the strata. The estimated difference in proportions was presented with a two-sided 95% confidence interval (CI) and a one-sided p-value for non-inferiority. Non-inferiority was declared if p < 0.05. In addition to the non-inferiority assessment on an absolute scale for the primary outcome, we estimated the treatment effect on a relative scale more likely to be transportable to individual patients, using a logistic regression model adjusted for sex, age (as a linear covariate on the log-odds scale), level of anastomosis (chest or neck), comorbidity according to the American Society for Anesthesiology (ASA) score (≤2/>2), clinical tumour stage, and neoadjuvant treatment (yes/no). The adjusted odds ratio was presented with a two-sided 95% CI and a two-sided p-value. A per protocol analysis was then performed where only patients with no NG tube treatment constituted the intervention per protocol group and those who reached at least the five days NG tube treatment the control per protocol population.

For the secondary outcomes, pneumonia was analysed with a logistic regression model, length of hospital stay and length of stay in high dependency unit with linear regression models and overall complications were analysed using a proportional odds logistic regression model, all adjusted for the same variables as for the primary endpoint, on the corresponding scale. 30- and 90-day mortality were analysed with Pearson’s chi squared test. All secondary outcomes were analysed without adjustment for multiplicity and the estimates was presented with two-sided 95% CIs and two-sided p-values.

Unadjusted models were used as sensitivity analyses. To assess the proportional odds assumption for complications, we fitted a binary logistic regression model for all the dichotomisations except for (≤4b/>4b), that could not be performed due to zero patients having a Clavien Dindo score of 5 in the group randomised to NG tube.

Subgroup analyses of the primary endpoint were performed by introducing subgroup indicators (if not already included) and a treatment subgroup interaction term in the logistic regression model, excluding any patients not possible to classify. These subgroups were age (above/below 70 years), sex (male/female), ASA score (≤2/>2), level of anastomosis (chest/neck), pyloroplasty (no/yes) and jejunostomy (no/yes). A separate subgroup analysis for centre was also performed. Estimates of relative treatment differences was presented as subgroup-specific odds ratios with 95% CIs and interaction p-values.

All analyses were performed with the statistical package R version 4.4.1.

#### Ethics committee approval

This trial was conducted after ethical approval in relevant authorities in Norway: Nr 256722, Sweden: Dnr 2021-03761, Finland: dnr 85/2021/266§ and Denmark Jnr H21069333.

#### Role of the funding source

This trial was funded by the Swedish Cancer Society and the Nordic Cancer Union. The funder of the study had no role in study design, data collection, data analysis, data interpretation, or writing of the report.

## Results

A total of 564 patients were screened for eligibility and 448 patients (88%) were randomised between January 21st 2022 and March 27th 2024. Intention to treat analyses were performed on these 448 patients whereof 217 were randomized to immediate NG-tube removal and 231 to control, Fig. 1. The majority of patients were male (n = 367, 81.9%), the mean age was 67.5 years, neck anastomosis was performed in 79 patients (17.6%), and the majority of patients had locally advanced disease (T3-T4), Table 1.

In the group randomised to NG tube, 95 patients did not complete the planned five days of conduit decompression due to patient refusal or accidental dislodgement of the NG tube, Fig. 1. In the group randomised to no NG tube, 9 patients had a tube inserted because of bloating or other signs of incomplete conduit emptying, Fig. 1. Complete data was obtained for all patients.

Overall, 83 patients (18.5%) had a postoperative anastomotic leak. In the intervention group (no NG tube) 48 patients (22.1%) experienced anastomotic leak compared to 35 (15.2%) in the control group. Non-inferiority was not established with a difference leak proportion to the advantage of NG tube use of −7.0% (95% CI −14.4%, 0.00%) p_non-inferiority_ 0.30. Eleven of the leakages (13.3%) were treated medically or with dietary modification only (Grade I), while 56 (67.5%) were treated with endoscopic measures (stent or vacuum therapy) and/or interventional radiology (Grade II) and 10 (12%) were reoperated (Grade III). The grading of leakage was similar between the groups, Table 2.

**Table 2:** Primary and secondary outcomes in the intention to treat population.

	No NG tube (n = 217)	NG tube five days (n = 231)
Anastomotic leak	48 (22.1%)	35 (15.2%)
Leak type		
Grade I	5	6
Grade II	31	25
Grade III	6	4
No data	6	
Any complication (CD > 2)	96 (44.2%)	91 (39.4%)
Pneumonia	52 (24.0%)	44 (19.0%)
Complication grade, CD		
0	60 (27.6%)	68 (29.4%)
1	15 (6.9%)	13 (5.6%)
2	46 (21.2%)	59 (25.5%)
3a	38 (17.5%)	47 (20.3%)
3b	36 (16.6%)	26 (11.3%)
4a	12 (5.5%)	14 (6.1%)
4b	7 (3.2%)	4 (1.7%)
5	3 (1.4%)	0 (0.0%)
Length of stay, days (95% CI)	18.0 (8–46)	16.4 (8–42)
Length of stay HD unit (95% CI)	4.50 (0–37)	3.62 (0–38)
30 day mortality	3 (1.4%)	0
90 day mortality	9 (4.1%)	6 (2.60%)

Absolute values for primary and secondary outcomes. Leak type according to Low et al.^14^ (Grade I no clinical manifestation, Grade II Endoscopic or interventional radiology, Grade III reoperation), CD: Clavien-Dindo classification for surgical complications,^15^ HD: High Dependency unit, i.e., all levels of care above ward.

Overall complications (Clavien-Dindo >2) occurred in 95 patients (43.8%) in the intervention group and 91 patients (39.4%) in the control group. Postoperative pneumonia occurred in 52 patients (24%) in the intervention group and in 44 (19%) patients in the control group respectively (p = 0.21). Correspondingly, overall 30- and 90-day mortality was 3 (0.67%) and 15 (3.3%) respectively with no significant differences between the treatment arms, Table 2. Of the 15 patients who died within 90 days six had an anastomotic leak (whereof five in the intervention group), Table 3.

**Table 3:** Complications requiring intervention (Clavien-Dindo >2) in both arms.

	No NG tube (n = 217)	NG tube five days (n = 231)
Complication grade	96 (44.2%)	91 (39.4%)
Grade 3a (Intervention required)	38 (17.5%)	47 (20.3%)
Pleural fluid	19	24
Anastomotic leak	5	6
Pulmonary embolism	3	1
Chyle leak	2	3
Pneumonia	3	2
Atrial fibrillation	1	1
Pneumothorax	1	1
Gastric outlet obstruction	2	6
Haematoma	1	0
Dysphagia	1	1
Wound dehiscence	0	1
Jejunostomy blocked	0	1
Grade 3b (Intervention in general anesthesia)	36 (16.6%)	26 (11.3%)
Anastomotic leak	17	17
Chylothorax	4	1
Paraconduit herniation	2	3
Brochial tear	1	0
Wound dehiscence	1	0
Gastric outlet obstruction	2	0
Pancreatic laceration	1	0
Dysphagia	1	0
Atrial fibbrilation	1	0
Haematoma	1	0
Sepsis	1	0
Small bowel obstruction	1	1
Respiratory failure	2	1
Missing	1	3
Grade 4a (Intensive care single organ failure)	12 (5.5%)	14 (6.1%)
Respiratory failure	6	8
Anastomotic leak	3	3
Pneumonia	1	1
Bleeding	1	1
Pulmonary embolism	1	0
Atrial fibrillation	0	1
Grade 4b (Intensive care multiorgan failure)	7 (3.2%)	4 (1.7%)
Airway fistula	4	0
Anastomotic leak	1	1
Sepsis	0	2
Chyle leak	1	0
Stroke	1	0
Epidural haematoma	0	1
Grade 5 (death) 30 days	3 (1.4%)	
Aspiration	2	0
Aortic rupture	1	0
Grade 5 (death) 31–90 days	6 (2.8%)	6 (2.60%)
Respiratory failure	2	0
Myocardial infarction	1	0
Sepsis	1	1
Early recurrance	2	1
Pulmonary embolism	0	1
Suicide	0	1
Heart failure	0	2

Postoperative complications in the two groups being base for the Clavien-Dindo classification.

**Fig. 2:**
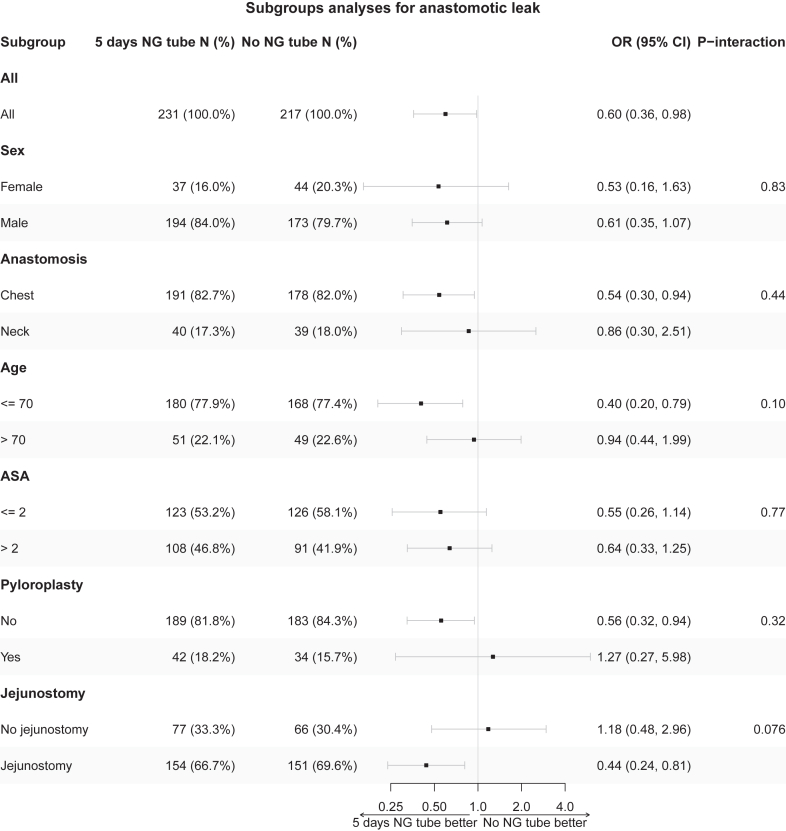
Subgroup analysis with test of the primary outcome anastomotic leak analysis using logistic regression. Treatment-subgroup interaction terms were introduced in the logit model; sex, age (as a linear covariate on the log-odds scale), level of anastomosis (chest or neck), comorbidity according to the American Society for Anesthesiology (ASA) score (≤2/>2), clinical tumour stage, and neoadjuvant treatment (yes/no).

The odds of anastomotic leak were lower in patients with a NG tube (adjusted odds ratio 0.60 (95% CI 0.36, 0.98) p = 0.043). In the analysis of secondary outcomes, including rates of pneumonia, no statistically significant differences, were found, Fig. 2, Table 4.

**Table 4:** Multivariate analysis of effect of NG tube in 448 patients undergoing oesophagectomy for cancer.

Outcome	Risk difference	95% Confidence interval	
Anastomotic leak	−7.0%	(−14.4%, 0.3%)	

	Odds Ratio		p-value

Anastomotic leak	0.60	0.36–0.98	0.043^a^
Pneumonia	0.73	0.45–1.16	0.18^a^
Complications	0.79	0.56–1.10	0.16^b^

	Mean difference		

Length of stay, days	−1.17	−4.03 to 0.55	0.14^c^
Length of stay HD unit	−1.03	−2.13 to 0.07	0.066^c^

Risk difference stratified on age and sex and multivariate analysis adjusted for sex, age (as a linear covariate on the log-odds scale), anastomosis (chest or neck), ASA score (≤2, >2) neoadjuvant treatment and pathological t-stage of primary and secondary outcomes.

a Logistic regression.

b Proportional odds logistic regression model.

c Linear regression model.

In the per-protocol analysis, the risk difference was −11.3% to the advantage of NG tube (95% CI, −19.1, −0.3%). The secondary outcomes were in line with the intention to treat analysis and in the adjusted analysis, the length of stay in high dependency unit was also significantly longer in the intervention group (no NG-tube), Supplementary Tables S2 and S3.

No interactions between subgroups and treatment with NG tube could be shown, Fig. 2 and Supplementary Fig. S1. The sensitivity analyses showed similar results as the main analysis, Supplementary Tables S4 and S5.

In the 95 patients of the control group with less than 5 days of NG tube drainage, 20% had anastomotic leak, 21% pneumonia and 37% Calvien-Dindo >2, Supplementary Table S6.

## Discussion

This multicentre randomised controlled trial could not establish non-inferiority for abstaining from postoperative NG tube use compared to the routine of five days NG tube decompression of the gastric conduit after oesophagectomy for cancer. In contrast, we observed a lower proportion of anastomotic leaks in patients randomised to routine use of NG tube drainage when adjusting for pre-specified risk factors.

Oesophageal cancer surgery has been associated with high risk and serious complications since its inception in the beginning of the 20th century. Advances in surgical technique and perioperative care has brought perioperative mortality rates from above 30% even in the best hands to around two percent in modern real-world practice.^17^^,^^18^ This history of high risk has cemented opinions in postoperative routines and the need for high quality trials for modifications of established practice is thus high.

In 2009, Dargraei et al. published a randomised controlled trial including 40 patients and found more anastomotic leaks in patients with NG tube.^9^ The same year a retrospective analysis of 124 patients concluded that NG tube use could be safely omitted.^10^ The authors reported three serious perforations while placing the NG tube indicating potential for risk avoidance by omitting the NG tube in routine practice. This report of iatrogenic NG tube injuries seems surprisingly high compared to our and other current data and today, the NG tube is generally placed under direct vision during the construction of the oesophagogastric anastomosis. In 2012, In a randomised controlled trial by Mistry and coworkers 150 patients had either “early removal” within 48 h or standard care with NG tube 6–10 days. They found no difference between the groups and concluded that early removal does not increase pulmonary or anastomotic complications.^12^ Another randomised controlled trial of early (day 1) versus late (day 7) NG tube removal including 71 patients concluded that this could be done safely, but had even lower event rates, thus facing the same methodological concerns. A recent propensity-score matched study including 438 patients found no difference in anastomotic leak rates or pneumonia with or without NG tube and recommend omission of its use.

A main driver for modifying existing common practice of NG tube decompression post oesophagectomy is the frequently reported patient discomfort with having the NG tube for several days. There is also a trend after gastrointestinal surgery in general towards simplification of postoperative routines by using less drains, no NG tube and employing early mobilisation and oral nutrition. This has been successful in many areas to great benefit for patients, e.g., after hepatectomy and pancreatectomy.^19^^,^^20^ Oesophageal surgery has been the last stand for NG tube drainage and ERAS guidelines advocate early removal when appropriate, but not omission.^21^ The hope of safely abandoning the use of post-oesophagectmy NG tube has been high. However, the shortcomings of many of the available trials, and the discrepancies in opinions in favour and against NG tube use spurred us to launch the present trial with the intention to provide solid non-inferiority before modifying the practice of NG tube gastric conduit decompression. In light of the evidence at hand we chose total omission of NG tube as the intervention. Our results prompt reflection and warrants caution before adoption of this new routine. A similar trend of simplification of postoperative care, i.e., omission of abdominal drainage tubes after gastrectomy, has recently been challenged in a randomised trial demonstrating benefit of drains.^22^

The anastomotic leak-rate of 18.5% is in line with previous reports from the Nordic countries.^23^ However, the 15% leak rate in the control arm, albeit higher figure compared to some trials, might be explained by vigilant reporting in a trial setting with high accrual rate (real-world data) and it is in line with results from the Swedish national registry.^17^ In the present trial, a routine and objective investigation with CT seven days after surgery of all patients revealed 11 (13.2%) sub-clinical leaks. In addition, the 30- and 90-day mortality (0.67 and 3.3% respectively) were low in an international context and did not differ between the ITT groups demonstrating good competence regarding handling of complications at the trial centres. This also applies to the similar rates between the trial arms of complications demanding intensive care.

The mechanism by which the risk difference in anastomotic leak arises is not evident but increased pressure in the gastric conduit leading to an increased tension in the anastomosis and potentially impeded gastric conduit wall blood circulation are potential mechanisms. In a study focused on gastric conduit emptying, 30.4% had partial conduit emptying and 7.1% delayed gastric emptying three days after oesophagectomy.^24^ This may contribute to increased pressure in the gastric conduit.^25^ This is also in line with lower anastomotic leak rates in patients who underwent preoperative pyloroplasty (Fig. 2). Another potential mechanism for our findings is that a gastric conduit filled with fluid might exert a pulling force on the anastomosis due to gravitational effect. Although tension on the anastomosis is challenging to measure, a lower ratio of gastric conduit length to thorax length has recently been associated to higher risk of anastomotic leak supporting this idea.^26^

In this trial, we focused on the research question of intention to treat with postoperative NG tube across all centres in their current practice. Other perioperative procedures were not standardised. There may therefore be differences in surgical technique, postoperative routines and other procedures across centres that could be relevant for the study and our observations. We did, however, stratify for centre in our randomisation. A substantial subset of patients (41.1%) allocated to the control arm did not complete the full five days of NG tube use (Fig. 1). Although most of these patients had the NG tube the first postoperative days the observation underlines challenges related to the use of NG tube after oesophagectomy. This may also have impacted study outcomes and from a speculative point of view the NG tube may be a stronger protective factor against anastomotic leak than demonstrated in this trial. Virtually all patients who are treated for an anastomotic leak received an NG tube and the small proportion who received NG tube for bloating or delayed gastric emptying are too few to justify separate analyses. In addition, these clinical signs and symptoms can be indications of an early leak too, further complicating the causal disentanglement. One might argue that the non-inferiority level of 9% is wide in this context but even with such a high acceptance of difference between groups, we could not show non-inferiority for the new practice of omitting NG tube and therefore discourage abstaining from conduit decompression after oesophagectomy.

A limitation of the trial is that the per protocol analyses risk overestimating the effect of NG tube because early removal might be associated with an event free postoperative course. Further, the trial is also limited in that it does not say exactly how long the decompression should be undertaken, but the intention to use decompression seems to guard against the dreaded complication of anastomotic leak. The non-inferiority limit, chosen by using annual variations in leak rate in Sweden, might be considered wide and not reflecting clinical equipoise. The fact that non-inferiority could not be demonstrated despite this is, in our view, meaningful information for surgeons. The open-label design is something which cannot be ruled out to influence care or reporting of complications even if the likelihood for this is considered small. Knowledge among patients and ward staff of treatment arm is impossible to avoid in this trial because sham-tubes or the like would be non-sensical. However, the radiologists were not privy to randomisation arm when assessing the primary outcome anastomotic leak in the CT investigations on day seven. The male predominance makes conclusions in the female sub-group difficult. It reflects disease incidence however, and high accrual without any evident gender differences was achieved. Because of legal restraints, information on ethnicity is not possible to gather in this trial but randomisation was stratified on centre and we see no reason to believe that this issue will influence the results.

A major strength of our study is the short period of inclusion, the high follow-up rate, high accrual and the multicentre participation with 12 of 21 eligible centres for study inclusion in the Nordic countries participating. Further, the accrual rate of 79% should enable good representation of the routine practice in the participating study centres. We focused on a simple intervention and a follow-up routine in line with our standard clinical follow-up making it possible not to lose any patients. The use of objective CT evaluation with oral contrast seven days postoperatively, with the radiologist blinded to treatment allocation, is also a strength related to identifying primary outcome events. The intention to treat analyses and the strict focus on the use of NG, or not, without any other modifications of clinical practice or routines at the study centres align the trial findings better to clinical practice than a more standardised and idealised trial context.

In conclusion, in this largest randomised trial to date investigating the impact of NG tube decompression of the gastric conduit after oesophagectomy, we could not conclude non-inferiority of abstaining from postoperative NG tube. In addition, the Supplementary analyses suggests that this may even be harmful. Omission of post-oesophagectomy NG tube decompression is not supported.

## Contributors

JH prepared the first draft and managed the overall project. JH, TM, MA and JK were national investigators and MN provided international coordination expertise. AN performed all statistical analyses of the data. All other authors provided data, reviewed results, provided guidance on methods, or reviewed and contributed to the Article.

## Data sharing statement

Source data cannot be shared according to the law in the countries including in this trial. Aggregated anonymized data can be obtained from the corresponding author on request.

## Declaration of interests

MS has received speakers honoraria from Novo Nordisk, paid to institution. JK has received grants from The Finnish Cancer Foundation, paid to institution. None of the other authors have competing interest related to this trial.

## Appendix A Supplementary data

Supplementary Tables

Clinical study protocol4.0_20250108

kiNETiC–DSMC report 2023
