# Supplementary material for: Nasogastric tube after oesophagectomy and risk of anastomotic leak: a Nordic, multicentre, open-label, randomised, controlled, non-inferiority trial
Source: Lancet Reg Health Eur. 2025 Jul 31;57:101411. doi: 10.1016/j.lanepe.2025.101411 (PMC12337195; doi:10.1016/j.lanepe.2025.101411)
Supplement: Supplementary Tables [file mmc1.docx]

Supplementary Table 1 Baseline characteristics for patients in the per protocol analysis

|  |  | No NG tube (n=208) | NG tube five days (n=136) |
| --- | --- | --- | --- |
| Sex | Male | 166 (79.8%) | 114 (83.8%) |
|  | Female | 42 (20.2%) | 22 (16.2%) |
| Age (SD, range) |  | 68·0 (10·0, 45-83) | 67·6 (9·3, 40-84) |
| ASA score | 1 | 13 (6.2%) | 4 (2.9%) |
|  | 2 | 108 (51.9%) | 74 (54.4%) |
|  | 3 | 83 (39.9%) | 58 (42.6%) |
|  | 4 | 4 (1.9%) | 0 (0.0%) |
| Smoking | Yes | 20 (9.6%) | 14 (10.3%) |
|  | No | 100 (48.1%) | 80 (58.8%) |
|  | Former | 84 (40.4%) | 42 (30.9%) |
|  | Unknown | 4 (1.9%) | 0 (0.0%) |
| Operation | Ivor Lewis | 172 (82.7%) | 116 (85.3%) |
|  | McKeown | 36 (17.3%) | 20 (14.7%) |
| Surgical access | Open surgery | 12 (5.8%) | 8 (5.9%) |
|  | Hybrid MIO | 79 (38.0%) | 50 (36.8%) |
|  | Total MIO | 117 (56·2 %) | 78 (57.4%) |
| Peroperative pyloroplasty | Yes | 32 (15·3%) | 24 (17·6%) |
| Clinical T stage | T1 | 5 (2.4%) | 5 (3.7%) |
|  | T2 | 35 (16.8%) | 28 (20.6%) |
|  | T3 | 146 (70.2%) | 93 (68.4%) |
|  | T4a | 22 (10.6%) | 10 (7.4%) |
| Clinical N stage | N0 | 104 (50.0%) | 73 (53.7%) |
|  | N1 | 57 (27.4%) | 31 (22.8%) |
|  | N2 | 40 (19.2%) | 26 (19.1%) |
|  | N3 | 7 (3.4%) | 6 (4.4%) |
| Clinical M stage | M0 | 203 (97.6%) | 129 (94.9%) |
|  | M1 | 5 (2.4%) | 7 (5.1%) |
| Neoadjuvant treatment | None | 33 (15·9%) | 25 (18·4%) |
|  | Radiochemotherapy | 55 (26·4%) | 45 (33·1%) |
|  | Chemotherapy | 120 (57·7%) | 66 (48.5%) |

Supplementary table 2 Primary and secondary outcomes in the per-protocol population

|  |  | No NG tube (n=208) | NG tube five days (n=136) |
| --- | --- | --- | --- |
| Anastomotic leak |  | 48 (23·1%) | 16 (11·8%) |
| Leak type | Grade I | 5 | 4 |
|  | Grade II | 31 | 11 |
|  | Grade III | 6 | 1 |
|  | No data | 6 |  |
| Any complication (CD>2) |  | 89 (42·8%) | 56 (41·2%) |
| Pneumonia |  | 47 (22·6%) | 24 (17·6%) |
| Complication grade, CD | 0 | 58 (27·9%) | 40 (29·4%) |
|  | 1 | 15 (7·2%) | 9 (6·6%) |
|  | 2 | 45 (21·6%) | 31 (22·8%) |
|  | 3a | 36 (17·3%) | 34 (25·0%) |
|  | 3b | 33 (15·9%) | 13 (9·6%) |
|  | 4a | 11 (5·3%) | 6 (4·4%) |
|  | 4b | 7 (3·4%) | 3 (2·2%) |
|  | 5 | 3 (1·4%) | 0 (0·0%) |
| Length of stay, days (95 % CI) |  | 17·7 (12·9) | 16·8 (10·1) |
| Length of stay HD unit (95 % CI) |  | 3·1 (4·79) | 4·29 (5·97) |
| 30 day mortality |  | 3 (1·4%) | 0 |
| 90 day mortality |  | 9 (4·3%) | 3 (2·21%) |

Supplementary table 3. Multivariate per protocol analysis of effect of NG tube in 344 patients undergoing oesophagectomy for cancer

| Outcome |  | Risk difference | 95% Confidence interval |  |
| --- | --- | --- | --- | --- |
| Anastomotic leak |  | -11·3% | (-19·1%-3·0%) |  |
|  |  | Odds Ratio |  | p-value |
| Anastomotic leak |  | 0·42 | 0·21-0·77 | 0·007* |
| Pneumonia |  | 0·70 | 0·40-1·22 | 0·22* |
| Complications |  | 0·80 | 0·54-1·18 | 0·27β |
|  |  | Mean difference |  |  |
| Length of stay, days |  | -1·09 | -3·67-1·48 | 0·40δ |
| Length of stay HD unit |  | -1·36 | -2·56-(-0·17) | 0·026δ |

Supplamantary Table 3. Risk difference stratified on age and sex. Multivariate analysis adjusted for sex, age (as a linear covariate on the log-odds scale), anastomosis (chest or neck), ASA score (≤ 2, > 2) neoadjuvant treatment and pathological t-stage of primary and secondary outcomes. *Logistic regression βProportional odds logistic regression model δ Linear regression model

Supplemenmtary table 4 Analysis of complication dichotomizations in the intention to treat population

| Outcome |  | Odds Ratio | 95% Confidence interval | p-value |
| --- | --- | --- | --- | --- |
| CD (≤0/> 0) |  | 0.89 | 0.58, 1.36 | 0.58 |
| CD (≤1/>1) |  | 0.94 | 0.63, 1.41 | 0.77 |
| CD (≤2/2) |  | 0.79 | 0.54, 1.16 | 0.24 |
| CD (≤3a/3) |  | 0.60 | 0.37, 0.95 | 0.030 |
| CD (≤3b/3b |  | 0.66 | 0.33, 1.31 | 0.24 |
| CD (≤4a/4a) |  | 0.40 | 0.11, 1.23 | 0.13 |

Supplementary Table 4. Complications fitted with a binary logistic regression model for all the dichotomisations except for (≤ 4b/> 4b), that could not be preformed due to no patients having a Clavien Dindo score of 5 in the group randomised to NG tube)*.* Intention to treat population.

Supplementary Table 5 Unadjusted analyses in the intention to treat population

|  |  | Odds Ratio | 95% Confidence interval | p-value |
| --- | --- | --- | --- | --- |
| Anastomotic Leakage |  | 0.63 | 0.39, 1.01 | 0.059 |
| Pneumonia |  | 0.75 | 0.47, 1.17 | 0.21 |
| Length of stay |  | -1.62 | -3.89, 0.66 | 0.16 |
| Length of stay in HD |  | -0.88 | -1.99, 0.22 | 0.12 |
| Clavien Dindo |  | 0.83 | 0.59, 1.15 | 0.25 |

Supplementary Table 2. Unadjusted analyses of the main and secondary outcomes in the intention to treat population.

Supplementary table 6. Safety data for 95 patients in the control arm with NG tube less than 5days

|  |  | NG ube <5 days (n=95) |
| --- | --- | --- |
| Anastomotic leak |  | 19 (20 %) |
| Leak type | Grade I | 2 (11%) |
|  | Grade II | 14 (74%) |
|  | Grade III | 3 (16 %) |
| Any complication (CD>2) |  | 35 (37 %) |
| Pneumonia |  | 20 (21%) |
| Complication grade, CD | 0 | 28 (29 %) |
|  | 1 | 4 (4 %) |
|  | 2 | 28 (29 %) |
|  | 3a | 13 (14 %) |
|  | 3b | 13 (14 %) |
|  | 4a | 8 (8 %) |
|  | 4b | 1 (1 %) |
|  | 5 | 0 (0 %) |
| Length of stay, days (95 % CI) |  | 15·8 (6-64) |
| Length of stay HD unit (95 % CI) |  | 4·4 (0-28) |
| 30 day mortality |  | 0 (0 %) |
| 90 day mortality |  | 0 (0 %) |

Supplemetary Table 7 Interclass correlation coefficient per arm for the primary and secondary outcomes.

| Outcome | No NG tube | 5 days NG tube |
| --- | --- | --- |
| Anastomotic leak | 0·0020 | 0·0295 |
| Pneumonia | 0·0341 | 0·0090 |
| Clavien-Dindo | 0·0659 | 0·0238 |
| Length of stay | 0·0917 | 0·2115 |
| Length of stay HD | 0·1348 | 0·0988 |

Supplementary Figure 1

Subgroup analysis with test of the primary outcome anastomotic leak using logistic regression. Treatment-subgroup interaction terms in form of operationg center were introduced in the logit model. One center could not be calculated because of no events (leaks).
