# Supplementary material for: Nasogastric tube after oesophagectomy and risk of anastomotic leak: a Nordic, multicentre, open-label, randomised, controlled, non-inferiority trial
Source: Lancet Reg Health Eur. 2025 Jul 31;57:101411. doi: 10.1016/j.lanepe.2025.101411 (PMC12337195; doi:10.1016/j.lanepe.2025.101411)
Supplement: Clinical study protocol4.0_20250108 [file mmc2.pdf]

CLINICAL STUDY PROTOCOL

kiNETiC-a Randomized Controlled Trial- Ng-tube post-EsophagecTomy Complications

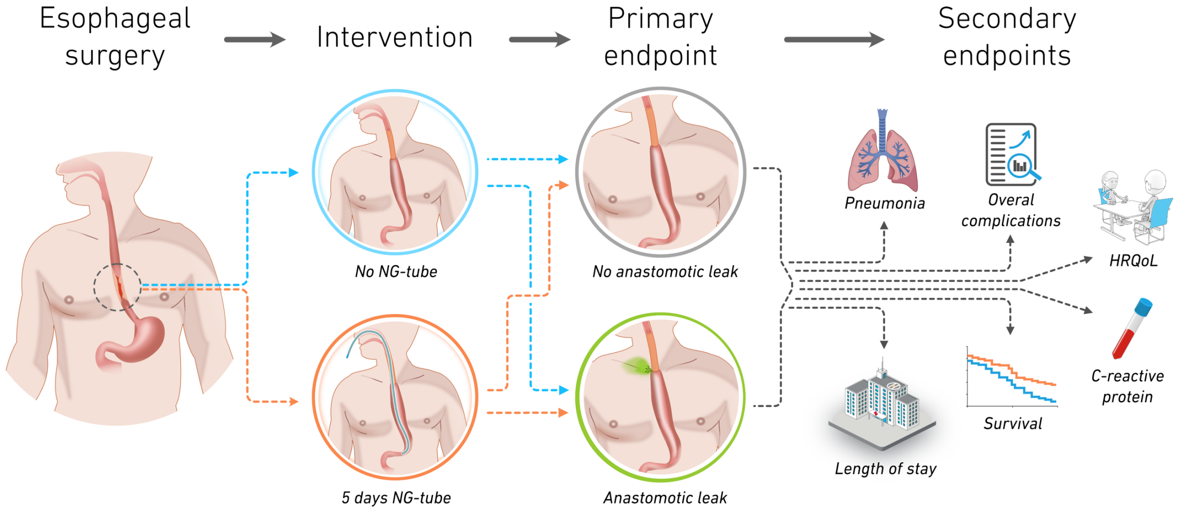

Clinical trials identifier:	ISRCTN39935085<br>https://doi.org/10.1186/ISRCTN39935085
Version Number:	4.0
Date:	2023-01-08
Research body:	Uppsala University
Principal Investigator:	Jakob Hedberg
Ethical reviewboard descision:	Norway 202-07-21: Nr 256722<br>Sweden 2021-10-12: Dnr 2021-03761<br>Finland 2021-12-01: dnr 85/2021/266§<br>Denmark 2022-06-27: Jnr H21069333

Contact addresses

Principal Investigator

Jakob Hedberg
Department of Surgical Sciences
Uppsala University
75185 Akademiska Sjukhuset
Uppsala
Sweden
Jakob.hedberg@surgsci.uu.se
+46186116507

Investigators

Fredrik Lindberg; Fredrik.Lindberg@regionvasterbotten.se
Magnus Sundbom; magnus.sundbom@surgsci.uu.se
Mats Lindblad; mats.lindblad@ki.se
Eva Szabo; eva.szabo@regionorebrolan.se
David Edholm; david.edholm@regionostergotland.se
Jan Johansson; jan.johansson@med.lu.se
Oscar Åkesson; oscar.akesson@skane.se
Per Löfdahl; per.lofdahl@vgregion.se
Eirik Kjus Aahlin; Eirik.Kjus.Aahlin@unn.no
Tom Mala; tommal@ous-hf.no
Hans-Olaf Johannessen; UXHOJO@ous-hf.no
Gjermund Johnsen; Gjermund.Johnsen@stolav.no
Lars Lundell; Lars-Lundell@ki.se
Michael Patrick Achiam; Michael.Patrick.Achiam.01@regionh.dk
Michael Bau Mortenssen; michael.bau.mortensen@rsyd.dk
Joonas Kauppila; joonas.kauppila@oulu.fi

To be expanded/amended as more centers join the trial

HRQOL Expert — Pernilla Lagergren; pernilla.lagergren@ki.se

Patient representative — Olof Riesenfeld representing PALEMA; olof@riesenfeld.org

International Coordinator — Magnus Nilsson; magnus.nilsson@ki.se

SYNOPSIS

Title: kiNETiC-trial – A Randomized Controlled Trial- Ng-tube post-EsophagecTomy Complications
Rational for the study: The mainstay of curative treatment of cancer in the esophagus and the gastroesophageal junction is surgical resection with a gastric conduit used for replacement of the resected esophagus. In Scandinavia, a naso-gastric tube (NG tube) is generally left in place after surgery but the clinical benefits and potential harms of this practice are unclear.
Study Design: A Randomized Controlled Trial
Study Population: Adult patients undergoing esophagectomy for cancer in Scandinavia
Number of patients: 450
Inclusion criteria:
Histopathologically confirmed esophageal cancer in locally advanced stages (cT1 N+ or cT2-4a any N; M0) and considered technically resectable by the local tumor board
Planned for esophagectomy with gastric conduit reconstruction
Age≥ 18 years
Written informed consent
Exclusion criteria
No resection performed (reason specified)
Other reconstruction than planned (Roux-limb/colon interposition)
Surgeon choosing to leave NG-tube (reason specified)
Patient unable to understand study information (including language difficulties)
Hypothesis
Abstaining from NG-tube use is non-inferior to using NG-tube after esophagectomy regarding anastomotic leak
Primary outcome variable
- Anastomotic leak type I-III according to Low et al.(1)

Secondary outcome variables
- Pneumonia according to Seesing et al.(2)
- Postoperative complications >3a according to Clavien-Dindo (3)
- Respiratory failure requiring invasive or non-invasive respiratory support
- C-reactive protein day 1-7
- Length of stay
- Length of ICU-stay
- Reintervention with nasogastric tube decompression
- Vomiting
- Overall survival
- Endoscopic appearance of anastomosis day 7(optional)
- PROM at discharge (qualitative, optional)

Study period: January 1st 2022 to December 31st 2025

TABLE OF CONTENTS

1. INTRODUCTION **7**
1.1 Background 7
1.2 Rationale for conducting the study 7
1.3 Risk/benefit evaluation 7

2. STUDY HYPOTHESIS, OBJECTIVES AND ENDPOINTS **8**
2.1 Hypothesis 8
2.2 Primary objective 8
2.3 Secondary objective(s) 8

3. STUDY DESIGN AND PROCEDURES **8**
3.1 Overall study design and flow-chart 8

Follow-Up **9**

Analysis **9**

Enrollment **9**

Allocation **9**
3.2 Rationale for study design 10
3.3 Study visits 10

4. STUDY POPULATION **10**
4.1 Inclusion criteria 10
4.2 Exclusion criteria 10
4.3 Subject enrollment and randomization 10
4.4 Discontinuation and withdrawal from study 10
4.5 Premature termination of study 10

5. STUDY INTERVENTION **11**
5.1 Intervention implementation 11
5.2 Control implementation 11
5.3 Blinding 11
5.4 Randomization strategy 11

6. STUDY MEASUREMENTS AND VARIABLES **11**
6.1 Primary outcome variable 11
6.2 Secondary outcome variable(s) 11

7. STATISTICS **12**
7.1 Sample size calculation 12
7.2 Prespecified subgroup analyses 12
7.3 Statistical analysis 12

8. DATA MANAGEMENT **13**
8.1 Recording of data 13
8.2 Data storage and management 13

9. QUALITY CONTROL AND QUALITY ASSURANCE **13**
9.1 Audits and inspections 14

10. Patient and Public Involvement ("Brukarmedverkan") **14**

11. ETHICS **14**
11.1 Ethics committee 14
11.2 Informed consent 14
11.3 Subject data protection 14
11.4 Insurances 14

12. PROTOCOL DEVIATIONS AND AMENDMENTS **15**

13 REPORT AND PUBLICATIONS **15**

14. STUDY TIMETABLE **15**
14.1 Study period 15
14.2 Definition of “End of study” 15

REFERENCES **15**

LIST OF ABBREVIATIONS

1. INTRODUCTION

1.1 Background

Esophageal cancer and cancer of the gastroesophageal junction (Siewert I and II), henceforth referred to as GEJ cancer, is the eight most common type of cancer globally and the incidence rate is rising (4, 5). The poor 5-year survival of around 10% makes esophageal cancer the seventh cancer globally ranked by years of life lost(6).

For local and locally advanced tumors, surgical resection gives the best chance for cure. Esophagectomy is one of the most demanding procedures in gastrointestinal surgery, with significant morbidity and a six per cent 90-day mortality even after centralization to University hospitals. After resection of the esophagus and gastroesophageal junction, the continuity of the gastrointestinal tract is usually reconstructed with a gastric conduit, created along the major curvature of the stomach, whereafter the esophagogastric anastomosis is performed in the chest or neck depending on tumor site.

The anastomosis and gastric conduit are traditionally drained with a nasogastric tube with a typical output of 100 to 300 milliliters daily(7). There are, however, significant downsides associated with the use of a nasogastric tube. First, it can be questioned how much of the accumulated fluid that actually is drained through the tube. Second, the tube has been shown to facilitate reflux in oral direction over the anastomosis and generate micro aspiration into the respiratory tract. Moreover, treatment with a nasogastric tube is often very troublesome for the patient(8, 9) and in many other fields of gastrointestinal surgery, routine nasogastric tube placement has been abandoned. All told, the evidence in favor of routine use of nasogastric tube in association with complex surgery of the upper GI tract is meagre (10).

1.2 Rationale for conducting the study

The benefits of nasogastric tube decompression after esophagectomy are largely unknown. Several downsides are known and no adequately powered trial has been conducted to date. In the well-established Scandinavian network for esophageal cancer surgery, the research question regarding the need for nasogastric tube after esophagectomy, is possible to answer. If non-inferiority regarding complications when abstaining from nasogastric tube is demonstrated, significant benefits can be gained, especially regarding the patients experience of surgical treatment(11).

1.3 Risk/benefit evaluation

Nasogastric tube decompression is standard of care today and the underlying idea is that it might prevent and treat gastric stasis and over-distention and thus, anastomotic complications and aspiration in the postoperative period. However, the main downsides of these tubes are risk for micro-aspiration and respiratory complications and above all great discomfort for the patient. In recent years, introduction of fast-track recovery programs after surgery have been adapted for several procedures, including esophagectomy(12). In these programs, early oral nutrition has been advocated as well as early removal of nasogastric tube. Possible benefits include decreased discomfort for the patients, possible decreased risk of micro-aspirations and early mobilization, as shown in other types of abdominal surgery(13).

2. STUDY HYPOTHESIS, OBJECTIVES AND ENDPOINTS

2.1 Hypothesis

Abstaining from NG-tube use is non-inferior to using NG-tube after esophagectomy regarding anastomotic leak.

2.2 Primary objective

To determine if it is safe to refrain from routine nasogastric decompression after esophageal resection. The primary endpoint will be anastomotic leak as defined by Low et al(1).

2.3 Secondary objective(s)

To assess differences, if any, on the following secondary endpoints.

- Pneumonia according to Seesing et al.(2)
- Postoperative complications >3a according to Clavien-Dindo (3)
- C-reactive protein day 1-7
- Length of stay
- Length of ICU-stay
- Reintervention with nasogastric tube decompression
- Vomiting
- Overall survival

3. STUDY DESIGN AND PROCEDURES

3.1 Overall study design and flow-chart

The study is designed as a multicenter randomized controlled trial, and the outcome variables will be drawn from the study CRF as well as from other validated national registries. The study will not be blinded. The study is expected to be conducted between 2022 and 2029 (Inclusion period 2022- 2025).

kinetic-trial Flow Diagram

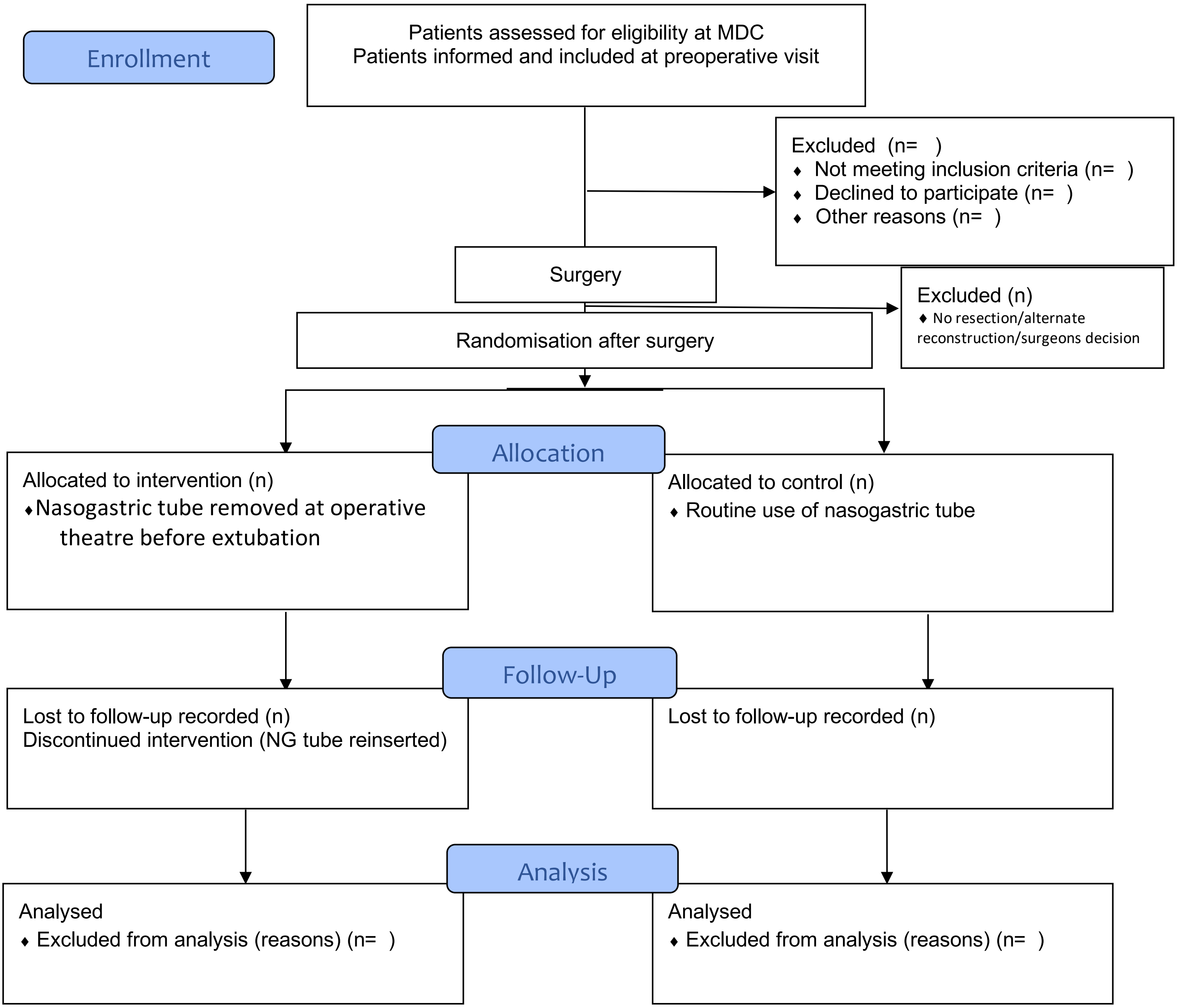

3.2 Rationale for study design

Esophageal resection is a rare operation with around 400 cases performed in Scandinavia annually. In order to make completion of the study possible within two to three years, the well-established Scandinavian trial network will be used. Leading surgeons from each center are represented in the study group and thus, knowledge gained will be incorporated in clinical use. Baseline data on participants is accessible from national quality registers at the time of randomization and all follow-up needed will be collected on the normal four to six weeks visit postoperatively.

3.3 Study visits

No extra study visits are planned for this study. The mandatory x-ray investigation on postoperative day seven (if no leak is previously diagnosed) is in line with postoperative routine in many centers. Long term survival and morbidity data will be collected from other registries.

4. STUDY POPULATION

4.1 Inclusion criteria

- Histopathologically confirmed esophageal or GEJ cancer in locally advanced stages (cT1a N+ or cT1b-4a any N; M0) and considered technically resectable by the local tumor board
- Age ≥ 18 years
- Planned for esophagectomy with gastric conduit reconstruction
- Written informed consent

4.2 Exclusion criteria

- No resection performed (reason specified)
- Alternative reconstruction method used (Roux-limb/colonic interponate)
- Surgeon choosing to leave NG-tube (reason specified)
- No ability to understand the study in terms of risk and benefits (including language difficulties)

4.3 Subject enrollment and randomization

Subject eligibility will be assessed by MDT conference. The operating surgeon will inform the patient of the study no later than the pre-operative visit where enrollment will take place. If the patient chooses to participate, informed consent will be signed and stored locally. The randomization will be performed after completion of the operation.

4.4 Discontinuation and withdrawal from study

All subjects are free to discontinue their participation in the study at any time without any consequence for their treatment or follow-up. Existing study data on that patient will be kept or destroyed depending on the patient’s preference.

4.5 Premature termination of study

The study group can stop the inclusion at any time and in that case, prompt information to the ethics board should be provided.

5. STUDY INTERVENTION

5.1 Intervention implementation

The clinical routine is to leave the nasogastric tube in place after esophagectomy. If patients are randomized to "no nasogastric tube", the surgeon removes it before waking of the patient in the operating theatre. All patients are given proton pump inhibitor according to local routines. Oral feeding and use of feeding jejunostomy will take place according to local routines.

5.2 Control implementation

Patients not randomized to nasogastric tube removal will have a tube in place the first five postoperative days. The tube will be of a diameter of no less than Ch16 and continuous or intermittent active or siphon suction will be applied (depending on local practice). Return from the drains will be recorded in line with local routine. The nasogastric tube will be removed prior to the chest CT (including peroral contrast) on day seven except in cases where the output exceeds 300 ml (+any ingested fluid) per 24 hours or the responsible clinician deems continued nasogastric decompression indicated. All patients are given proton pump inhibitor according to local routines. Oral feeding and use of feeding jejunostomy according to local routines.

5.3 Blinding

The study will not include any blinding procedures or "sham-tubes". The patient's allocation will be evident for all health care personnel. The radiologist assessing the day seven-Chest-CT will, however, be blinded since the nasogastric tube will be removed before this CT.

5.4 Randomization strategy

A randomization module will randomize in a 1:1 ratio between nasogastric tube or no nasogastric tube. For randomization, permuted block randomization with stratification for sex and neoadjuvant treatment (y/n), anastomosis site (neck or thorax) and center will be used.

6. STUDY MEASUREMENTS AND VARIABLES

6.1 Primary outcome variable

The primary outcome in this RCT will be anastomotic leakage according to Low et al.(1)

6.2 Secondary outcome variable()

Secondary outcome variables will be

- Pneumonia according to Seesing et al.(2)
- Postoperative complications >3a according to Clavien-Dindo (3)
- Respiratory failure requiring invasive or non-invasive respiratory support
- C-reactive protein day 1-7
- Length of stay
- Length of ICU-stay
- Reintervention with nasogastric tube decompression
- Vomiting

- Overall survival
- Endoscopic appearance of anastomosis day 7 (optional, appendix 1)
- PROM at discharge (qualitative, optional, appendix 2)

7. STATISTICS

7.1 Sample size calculation
In recent Scandinavian and Dutch trials, anastomotic leak was present in 18% of the patients and those without nasogastric tube did not have more (the studies were not powered for this question however)(14, 15).

For decision on non-inferiority level, the variations in the number of leaks in the Swedish register over the recent decade was examined and the variation between 10 and 22% led to choosing a non-inferiority threshold of 9%. With a power (β) of 80% and a significance (α) of 5%, this would require 216 patients in each group or 432 patients in total.

With some expected drop-outs (e.g., no resection performed due to more advanced disease than anticipated), a total of 450 patients will be randomized.

7.2 Prespecified subgroup analyses
Predefined subgroups to be analyzed include gender (male/female), neck anastomosis (y/n), early oral feeding (before CT day 5, y/n), age >75 (y/n), pyloric drainage procedure (y/n) and ASA and Clinical frailty score.

7.3 Statistical analysis
Baseline characteristics will be described by randomized treatment and in total. Categorical data will be described as total number and percentage, with missing data as a separate category. Numerical data will be described using number of patients with data, and median, quartiles, arithmetic mean and standard deviation for patients with data.

"Statistical testing" comparing the randomized treatment groups will be performed using chi- square tests for categorical variables and Wilcoxon's test for numerical data, using observed cases. The result will be presented as p-values and used only if needed to satisfy a non-CONSORT journal house style that requires Table 1 p-values. Since the groups are randomized, all perceived differences will be due to chance.

All outcomes will be analyzed using the intention to treat (ITT) principle where patients randomized to a certain group will be followed irrespective of the actual treatment, offering unbiased assessments of treatment efficacy(16). The primary outcome anastomotic leak will be analyzed with a logistic regression model adjusted for sex, age (as a linear covariate on the log-odds scale) and level of anastomosis (chest or neck). Subgroup analyses will be performed by introducing (or substitute if term already included) a treatment- subgroup interaction term in the logit model, excluding any patients not possible to classify. Estimates of treatment differences will be presented with odds ratios and two-tailed 95% confidence intervals and associated p-values. A two- tailed P-value of <.05 is considered

statistically significant. Secondary outcomes will be analyzed without adjustment for multiplicity.

7.4 Statistical amendment

Additional input from statistical expertise in conjunction with presentation of the study in the Journal Diseases of the Esophagus(17), has yielded slight adjustements in the statistical plan:

The primary objective, non-inferiority of the experimental arm (no NG tube), will be assessed overall and with stratified (women aged <70 years, women aged ≥70 years, men aged <70 years, and men aged ≥70 years) Miettinen–Nurminen two-sided 90% confidence interval (CI) for the difference in proportions and one-sided P-value for non-inferiority. Non-inferiority will be considered shown if P < 0.05 (one-sided).

Thus, dichotomization was adjusted from 75 to 70 years and Mittinen-Nurminen analysis was planned for the primary outcome. Early feeding was omitted from subgroup analyses as were ASA and clinical frailty scores due to definition weaknesses.

8. DATA MANAGEMENT

8.1 Recording of data

Swedish baseline and surgical data will be collected in a study database with anonymized data. An e-CRF will be used for all mandatory primary and secondary outcomes except survival. All study data will be transferred from the e-CRF to the study database. Additional outcome data (survival) will be accrued from national registries and added to the study database. Written consent will be stored at each study site and the local study participant are responsible for source documents being accessible for monitoring.

8.2 Data storage and management

All data will be recorded, handled and stored in a way that allows its accurate reporting, interpretation and verification. All source data, including a copy of the completed study database ant the original protocols with amendments will be stored at the department of Surgical Sciences at Uppsala University Hospital for a minimum period of ten years in accordance with Swedish law (Chapter 10, LVFS 2011:19).

When inclusion is complete, any deviations from the protocol will be recorded. When the study database has been declared complete and accurate, it will be locked and available for analysis.

9. QUALITY CONTROL AND QUALITY ASSURANCE

The principal investigator will arrange trial meetings in conjunction with the Scandinavian esophagogastric cancer (SEGCG) meetings. This will ensure that the local investigators are adequately informed about the protocol and that data input and storage are according to protocol.

9.1 Audits and inspections

A trial safety committee will be appointed to monitor the progress of the trial in terms of protocol adherence, patient safety and data safety. This committee consists of Professor Anders Wanhainen (Chair), Olof Wolf and Misha Luyer.
In addition, authorized members of the study group can make inspections at the sites included in the study. The purpose of these inspections is to ensure adherence to the study protocol. The local representative is responsible for secure storage of the original consent forms.

10. Patient and Public Involvement ("Brukarmedverkan")

The Swedish patient organization PALEMA support this trial. They have access to the latest version of this protocol and have a seat in the study steering committee.

11. ETHICS

11.1 Ethics committee

The final version of the study protocol and the informed consent will be provided and be given a favorable opinion in writing by the Ethics Committee. The principal investigator is responsible for providing any amendments to the protocol to the Ethics Committee in Sweden.

11.2 Informed consent

The local investigator at each site must ensure that each subject is given information about the possible risks and benefits of the study. The subject should be provided the opportunity to ask questions and allowed time to consider the information given. A clear notification that all subjects are free to withdraw from the study at any time shall also be given.

Written consent must be obtained before randomization and study intervention. Monitors must be granted access to relevant medical records, without violation the confidentiality of the subject. This is achieved by a separate consent form for monitoring from the subject or a legal representative, granting the monitor full access to medical records. The monitor is health care personnel bound by usual confidentiality rules and regulations.

The original consent form is stored locally and the subject is given one copy and one copy is sent to the principal investigator.

11.3 Subject data protection

Data will be stored in accordance with relevant data protection and privacy legislation. The consent form will include information concerning the data storage, including information that study monitors will have temporary access to hospital records for verification purposes.

11.4 Insurances

The subjects are covered by insurance in the form of the Swedish Patients Injury Act.

12. PROTOCOL DEVIATIONS AND AMENDMENTS

Any protocol amendments or adjustments to the information in the consent form(s) will be submitted to the Ethical Committee for approval before implementation. All changes to the protocol will be registered at the study database with information on the nature of the adjustment and the date for implementation.

13 REPORT AND PUBLICATIONS

Within one year after completion of the study, a study report, including publications and abstracts from the study, will be submitted to the Ethics Committee. After finalization of the study report, the results will be made publicly available at an open site for clinical trials (ISRCTN Registry).

Authors of publications must meet the International Committee of Medical Journal Editors guidelines for authorship and must satisfy the three criteria that follow:
a) Authors must make substantial contributions to the conception and design of the trial, acquisition of data, or analysis of data and interpretation of results.
b) Authors must draft the publication or, during draft review, provide contributions (data analysis, interpretation, or other important intellectual content) leading to significant revision of the manuscript with agreement by the other authors.
c) Authors must provide written approval of the final draft version of the publication prior to submission.
All contributors who do not meet these three criteria for authorship should be listed in an acknowledgments section within the publication, if allowed by the journal. Co-authorship will be offered any institution where ≥ 15 patients have been enrolled.

14. STUDY TIMETABLE

14.1 Study period

Estimated subject enrollment start: January 1st 2022
Estimated last enrollment (450 randomized): Dec 31st 2024
Last follow up (survival data): Dec 31st 2029

14.2 Definition of "End of study"

The end of study is defined as the last visit of the last patient included.

REFERENCES

1. Low DE, Alderson D, Cecconello I, Chang AC, Darling GE, D'Journo XB, et al. International Consensus on Standardization of Data Collection for Complications Associated With Esophagectomy: Esophagectomy Complications Consensus Group (ECCG). Ann Surg. 2015;262(2):286-94.
2. Seesing MFJ, Wirsching A, van Rossum PSN, Weijs TJ, Ruurda JP, van Hillegersberg R, et al. Defining pneumonia after esophagectomy for cancer: validation of the Uniform Pneumonia Score in a high volume center in North America. Diseases of the esophagus : official journal of the International Society for Diseases of the Esophagus / ISDE. 2018;31(6).

3. Clavien PA, Barkun J, de Oliveira ML, Vauthey JN, Dindo D, Schulick RD, et al. The Clavien-Dindo classification of surgical complications: five-year experience. Ann Surg. 2009;250(2):187-96.
4. Zhang Y. Epidemiology of esophageal cancer. World journal of gastroenterology. 2013;19(34):5598-606.
5. Torre LA, Bray F, Siegel RL, Ferlay J, Lortet-Tieulent J, Jemal A. Global cancer statistics, 2012. CA Cancer J Clin. 2015;65(2):87-108.
6. Global Burden of Disease Cancer C, Fitzmaurice C, Akinyemiju TF, Al Lami FH, Alam T, Alizadeh-Navaei R, et al. Global, Regional, and National Cancer Incidence, Mortality, Years of Life Lost, Years Lived With Disability, and Disability-Adjusted Life-Years for 29 Cancer Groups, 1990 to 2016: A Systematic Analysis for the Global Burden of Disease Study. JAMA Oncol. 2018;4(11):1553-68.
7. Zhao Y, Guo J, You B, Hou S, Hu B, Li H. The dynamic of nasogastric decompression after esophagectomy and its predictive value of postoperative complications. J Thorac Dis. 2016;8(Suppl 1):S99-S106.
8. Mistry RC, Vijayabhaskar R, Karimundackal G, Jiwnani S, Pramesh CS. Effect of short-term vs prolonged nasogastric decompression on major postesophagectomy complications: a parallel-group, randomized trial. Arch Surg. 2012;147(8):747-51.
9. Pan H, Hu X, Yu Z, Zhang R, Zhang W, Ge J. Use of a fast-track surgery protocol on patients undergoing minimally invasive oesophagectomy: preliminary results. Interact Cardiovasc Thorac Surg. 2014;19(3):441-7.
10. Weijs TJ, Kumagai K, Berkelmans GH, Nieuwenhuijzen GA, Nilsson M, Luyer MD. Nasogastric decompression following esophagectomy: a systematic literature review and meta-analysis. Diseases of the esophagus : official journal of the International Society for Diseases of the Esophagus / ISDE. 2017;30(3):1-8.
11. Hoffmann S, Koller M, Plaul U, Stinner B, Gerdes B, Lorenz W, et al. Nasogastric tube versus gastrostomy tube for gastric decompression in abdominal surgery: a prospective, randomized trial comparing patients' tube-related inconvenience. Langenbeck's archives of surgery / Deutsche Gesellschaft fur Chirurgie. 2001;386(6):402-9.
12. Jamel S, Tukanova K, Markar SR. The evolution of fast track protocols after oesophagectomy. J Thorac Dis. 2019;11(Suppl 5):S675-S84.
13. Cheatham ML, Chapman WC, Key SP, Sawyers JL. A meta-analysis of selective versus routine nasogastric decompression after elective laparotomy. Ann Surg. 1995;221(5):469-76; discussion 76-8.
14. Berkelmans GHK, Fransen LFC, Dolmans-Zwartjes ACP, Kouwenhoven EA, van Det MJ, Nilsson M, et al. Direct Oral Feeding Following Minimally Invasive Esophagectomy (NUTRIENT II trial): An International, Multicenter, Open-label Randomized Controlled Trial. Ann Surg. 2020;271(1):41-7.
15. Nilsson K, Klevebro F, Rouvelas I, Lindblad M, Szabo E, Halldestam I, et al. Surgical Morbidity and Mortality From the Multicenter Randomized Controlled NeoRes II Trial: Standard Versus Prolonged Time to Surgery After Neoadjuvant Chemoradiotherapy for Esophageal Cancer. Ann Surg. 2020;272(5):684-9.
16. Montori VM, Guyatt GH. Intention-to-treat principle. CMAJ. 2001;165(10):1339-41.
17. Hedberg J, Sundbom M, Edholm D, Aahlin EK, Szabo E, Lindberg F, et al. Randomized controlled trial of nasogastric tube use after esophagectomy: study protocol for

the kinetic trial. Diseases of the esophagus : official journal of the International Society for Diseases of the Esophagus / ISDE. 2024.
