# Supplementary material for: Nasogastric tube after oesophagectomy and risk of anastomotic leak: a Nordic, multicentre, open-label, randomised, controlled, non-inferiority trial
Source: Lancet Reg Health Eur. 2025 Jul 31;57:101411. doi: 10.1016/j.lanepe.2025.101411 (PMC12337195; doi:10.1016/j.lanepe.2025.101411)
Supplement: kiNETiC–DSMC report 2023 [file mmc3.pdf]

kiNETiC-a Randomized Controlled Trial- Ng-tube post-EsophagecTomy Complications

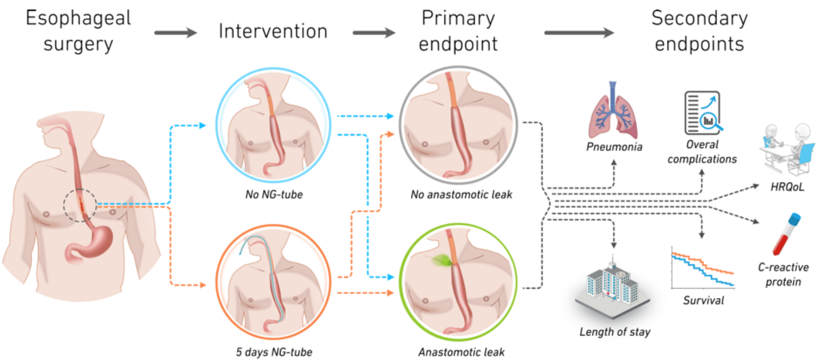

Data Safety and Monitoring Protocol

Appointed members

Anders Wanhainen, Uppsala, Sweden (chair)
Olof Wolf, Uppsala, Sweden
Misha Luyer, Eindhoven, The Netherlands

Background

The mainstay of curative treatment of cancer in the esophagus and the gastroesophageal junction is surgical resection with a gastric conduit used for replacement of the resected esophagus. In Scandinavia, a naso-gastric tube (NG tube) is generally left in place after surgery but the clinical benefits and potential harms of this practice are unclear. Therefore, a randomized controlled trial is underway in Sweden, Norway, Finland and Denmark. The data safety and monitoring committee (DSMC) plays a critical role in ensuring the safety and integrity of this trial. Key responsibilities of the DSMC include:

- Monitoring the progress of the study and reviewing data on an ongoing basis to identify any safety concerns or other issues that may arise.
- Providing guidance to the study team on how to address any issues or concerns that are identified.
- Reviewing adverse events and serious adverse events to assess their impact on the study and to determine whether any action is needed.
- Making recommendations to the study sponsor or principal investigator regarding the continuation, modification, or termination of the study, based on the data and safety concerns that are identified.
- Ensuring that the study is conducted in accordance with good clinical practices and that the rights and welfare of study subjects are protected.
- Providing regular updates to the study sponsor and other stakeholders on the progress of the study and any issues or concerns that have been identified

Meeting date:

2023-05-29

Adverse events/Serious adverse events

No serious unexpected adverse events have been reported to the trial steering committee 2022.

Recommendation (continuation/modification/interim analysis/termination of trial)

The study is carried out according to plan, without serious events or major deviations. The DSMC therefore recommends continuation of the study

Suggested modification if any

None

Uppsala 2023-05-29

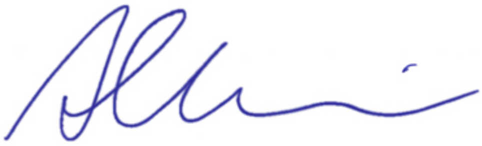

Anders Wanhainen, on behalf of the DSMC
